# Crystallographic Study of the LUMI Intermediate of Squid Rhodopsin

**DOI:** 10.1371/journal.pone.0126970

**Published:** 2015-05-29

**Authors:** Midori Murakami, Tsutomu Kouyama

**Affiliations:** 1 Department of Physics, Graduate School of Science, Nagoya University, Nagoya, Japan; 2 RIKEN Harima Institute/SPring-8, 1-1-1, Kouto, Mikazuki, Sayo, Hyogo, Japan; Zhejiang University, CHINA

## Abstract

Upon absorption of light, the retinal chromophore in rhodopsin isomerizes from the 11-*cis* to the *trans* configuration, initiating a photoreaction cycle. The primary photoreaction state, bathorhodopsin (BATHO), relaxes thermally through lumirhodopsin (LUMI) into a photoactive state, metarhodopsin (META), which stimulates the conjugated G-protein. Previous crystallographic studies of squid and bovine rhodopsins have shown that the structural change in the primary photoreaction of squid rhodopsin is considerably different from that observed in bovine rhodopsin. It would be expected that there is a fundamental difference in the subsequent thermal relaxation process between vertebrate and invertebrate rhodopsins. In this work, we performed crystallographic analyses of the LUMI state of squid rhodopsin using the P62 crystal. When the crystal was illuminated at 100 K with blue light, a half fraction of the protein was converted into BATHO. This reaction state relaxed into LUMI when the illuminated crystal was warmed in the dark to 170 K. It was found that, whereas *trans* retinal is largely twisted in BATHO, it takes on a more planar configuration in LUMI. This relaxation of retinal is accompanied by reorientation of the Schiff base NH bond, the hydrogen-bonding partner of which is switched to Asn185 in LUMI. Unlike bovine rhodopsin, the BATHO-to-LUMI transition in squid rhodopsin was accompanied by no significant change in the position/orientation of the beta-ionone ring of retinal.

## Introduction

Rhodopsin is the primary photoreceptor molecule in the visual signaling cascade. The activation process of rhodopsin is initiated by the photo-isomerization of the retinal chromophore from the 11-cis to all-trans configuration. The primary photoreaction state (BATHO) relaxes thermally through lumirhodopsin (LUMI) to an activated state, META, which in turn stimulates the heterotrimeric G protein to activate the enzymatic cascade in photoreceptor cells [1–4].

To date, bovine and squid rhodopsins have been crystallized and their structures have been determined at 2.2–2.8 Å resolutions [5–7]. The structural comparison of these proteins revealed that there is a profound difference in the structure of the retinal-binding pocket between vertebrate and invertebrate rhodopsins. In the resting state (RHOD) of squid rhodopsin, the plane of the retinal polyene chain is orientated parallel to the membrane with the Schiff base being hydrogen-bonded to either Asn87 in helix I and/or Tyr111 in helix III. The postulated counterion, Glu180 in the E-2 loop, is located 5 Å from the Schiff base. In bovine rhodopsin, on the other hand, the plane of the polyene chain is orientated perpendicular to the membrane, and the protonated Schiff base is salt-bridged to the counterion Glu113 in helix III. Crystallographic studies of the primary photochemical reaction in bovine and squid rhodopsins have also been carried out [8,9]. Upon formation of BATHO in squid rhodopsin, the central part of the retinal polyene chain undergoes a large rotation (approximately 90 degrees) with no significant movement of the β-ionone ring. In bovine bathorhodopsin, only limited movement in the retinal polyene chain has been observed.

The activation process of bovine rhodopsin has been extensively studied. From cross-linking studies of bovine rhodopsin, it was argued that significant movements of helices III and IV were induced upon the formation of LUMI [10]. This argument was supported by a recent crystallographic study of bovine lumirhodopsin, which showed that the large movement of the β-ionone ring of retinal is accompanied by a significant displacement of the middle moiety of helix III [11]. Recently, crystallographic analyses of Meta-II and opsin were reported for bovine rhodopsin [12–14]. Their structures suggested that a large outward movement of the cytoplasmic half of helix VI triggers binding of transducin to the cytoplasmic surface of rhodopsin.

Unlike vertebrate rhodopsin, the photoactive form of invertebrate rhodopsins is stable under physiological conditions and its META state is returned to the resting state by a second photon [1]. However, the details of the activation process of invertebrate rhodopsin have remained unclear. In this study, we performed a crystallographic study of the LUMI state of squid rhodopsin to elucidate the essential structural change occurring in the activation process.

## Results

### Trapping of LUMI

**Fig 1:**
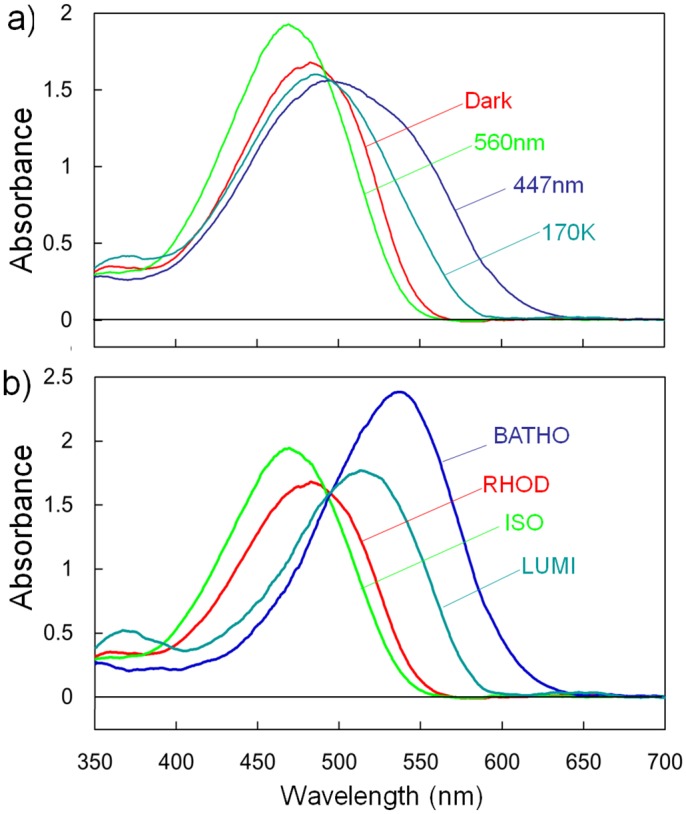
Absorption spectra of RHOD, BATHO, ISO, and LUMI in a frozen crystal at 100 K. (**a**) Red line: the absorption spectrum of a frozen crystal that was kept in the dark (Dark). Dark blue line: the spectrum recorded after one minute of illumination at 100 K with blue light at 447 nm (447nm). Yellowish-green line: the spectrum recorded after one minute of illumination at 100 K with yellow light at 560 nm (560nm). Cyan line: the spectrum recorded after the crystal was illuminated at 100 K with blue light, warmed in the dark to 170 K and re-cooled to 100 K (170 K). (**b**) Calculated absorption spectra of RHOD (red line), BATHO (blue line), ISO (green line) and LUMI (cyan line).

To investigate the thermal relaxation process from BATHO to LUMI, we measured adsorption changes induced upon warming of a single frozen crystal to various temperatures. Firstly, a dark-adapted crystal was flash-cooled with liquid propane and the spectrum was recorded at 100 K in the dark to determine the absorption spectrum of RHOD (Fig 1). The second spectrum was subsequently recorded after one minute of illumination of this crystal with blue light at 447 nm from a continuous wave laser (~1 mW/mm^2^) (Fig 1a, 460 nm). Under these illumination conditions, a photo-equilibrium state among three states (RHOD, BATHO and the artificial 9-cis isorhodopsin (ISO) was established; the contents of RHOD, BATHO and ISO were calculated to be 5%, 50% and 45%, respectively [9]. The third spectrum was recorded after one minute of illumination of the crystal with monochromatic yellow light at 560 nm, in which a photo-equilibrium state consisting of 98% ISO and 2% RHOD was established (Fig 1a, 560nm) [9]. The fourth spectrum was recorded after this crystal was illuminated at 100 K with blue light at 447 nm, warmed in the dark to 170 K and then re-cooled to 100 K at a rate of ~10 K/minute (Fig 1a, 170K). This absorption spectrum exhibited a negligibly small absorbance in the wavelength region above 600 nm. From this spectral feature, it was suggested that the full conversion of BATHO to LUMI occurred at 170 K. Since META was scarcely formed at this temperature, the content of LUMI after crystal warming to 170 K was estimated to be 50%.

The absorption spectrum of LUMI was obtained by subtracting the contributions of ISO and RHOD from the absorption spectrum of the crystal that was illuminated with blue light at 100 K, warmed in the dark to 170 K, and then re-cooled to 100K (Fig 1a, 170K). The calculated absorption spectrum of LUMI exhibits the absorption maximum at 514 nm (Fig 1b). A similar spectral feature was previously reported for LUMI generated in solubilized squid rhodopsin [15, 16].

### X-ray radiation-induced absorption changes in a frozen crystal containing LUMI

**Fig 2:**
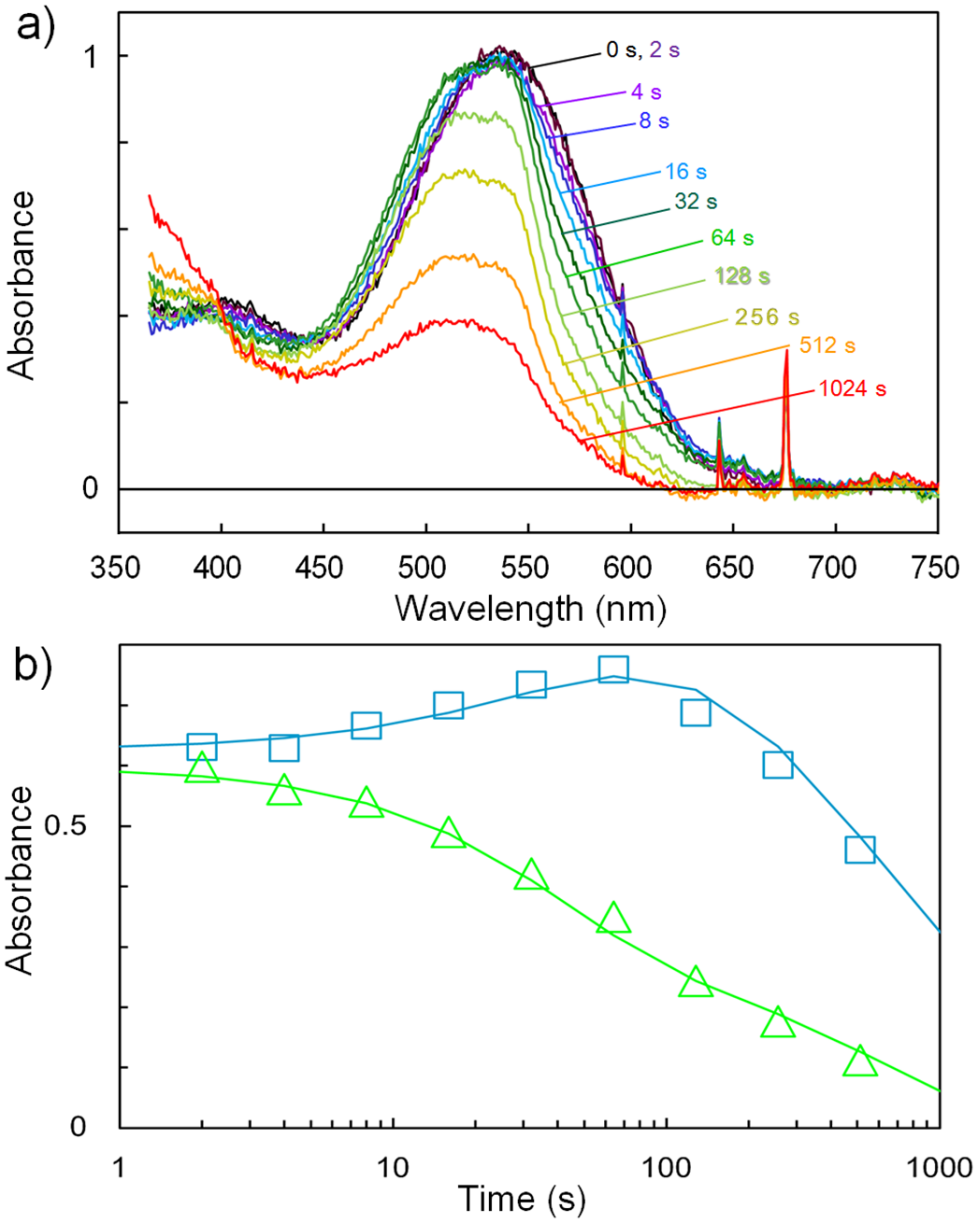
X-ray radiation-induced absorption changes observed at 100 K for the *P6*_*2*_ crystal consisting of 50% LUMI, 45% ISO and 5% RHOD. (**a**) Absorption spectra before (black line) and after exposure to monochromatic X-ray radiation at 1 Å with a flux rate of 4 ×10^12^ photons mm^-2^ sec^-1^ for various periods (colored lines). The numerals in this panel indicate the accumulated exposure time expressed in seconds. (Temperature drift in a Hg/Xe lamp used as the measuring light source caused artificial peaks at 645, 595 and 547 nm.) (**b**) Difference spectra derived by subtracting the absorption spectrum of the undamaged crystal from those observed after exposure to monochromatic X-ray radiation for various periods (colored lines). The numerals in this panel indicate the accumulated exposure time expressed in seconds. (**c**) Kinetics of X-ray radiation-induced absorption changes at 533 and 440 nm are shown as green squares, blue triangles and black crosses, respectively. The absorption changes were fitted by two exponential components (solid lines).

A previous crystallographic study of the M state of bacteriorhodopsin showed that a non-negligible amount of M was converted into a product with a 13-trans retro retinal by an X-ray flux of 2 × 10^15^ photons/nm^2^ [17]. To investigate the liability of LUMI to X-ray radiation, we investigated X-ray radiation-induced absorption changes in a frozen crystal containing LUMI and ISO as the major reaction states (50% and 47%, respectively); i.e., a frozen crystal (~0.1 mm) that had been illuminated at 100 K with blue light at 447 nm, warmed to 170 K and re-cooled to 100 K was exposed to synchrotron radiation (0.15 × 0.15 mm^2^) at a wavelength of 1 Å with a flux rate of 4 × 10^12^ photons/mm^2^/sec. It was observed that the absorbance at 530 nm decreased with the increasing exposure time and, instead, the absorbance at 440 nm increased (Fig 2a). When the exposure time was further increased, the absorption bands in the visible region slowly diminished and a new absorption band arose in the UV region. Our previous work showed that the mixture state of RHOD, ISO and BATHO was first modified into the orange X-ray products with absorption peaks at 420–490 nm [9].

In Fig 2b, the absorption changes observed at various wavelengths are plotted against the X-ray exposure time. The absorption decay kinetics at 533 nm, where the absorbance of LUMI is much higher than those of ISO and RHOD, were described with two time constants (40 s and 600 s). On the other hand, the absorbance at 440 nm increased with a time constant of 40 s and then decayed with a time constant of 600 s. This result suggests that the mechanism of the X-ray-induced modification of LUMI is not much different from that reported for bacteriorhodopsin [18]; namely, a fraction of LUMI was converted into an orange X-ray product upon exposure to an X-ray flux of ~1.6 × 10^14^ photons/mm^2^ and, after an dynamic equilibrium between LUMI and its first X-ray product was established, this equilibrium state was irreversibly converted into a colorless product upon exposure to a much higher flux of X-rays (~2.4 × 10^15^ photons/mm^2^).

### X-ray radiation-induced structural changes in a frozen crystal containing LUMI

We next investigated the structural changes that would take place when a frozen crystal containing LUMI was exposed to a low flux of X-rays. For this purpose, two crystals containing a similar amount of LUMI were mounted with different orientations and rotated at a speed of 1° per frame during diffraction data collection, which was performed using a synchrotron X ray beam at 1 Å and with a flux rate of 2 × 10^13^ photons/mm^2^ per frame. In the subsequent data reduction process, the diffraction data collected in an initial phase of X-ray exposure (i.e. the first 30 frames) were merged and the merged data (*F*_1st_) were compared with those (*F*_2nd_) collected in a late phase of the X-ray exposure (i.e., the 31st to the 60th frames).

**Fig 3:**
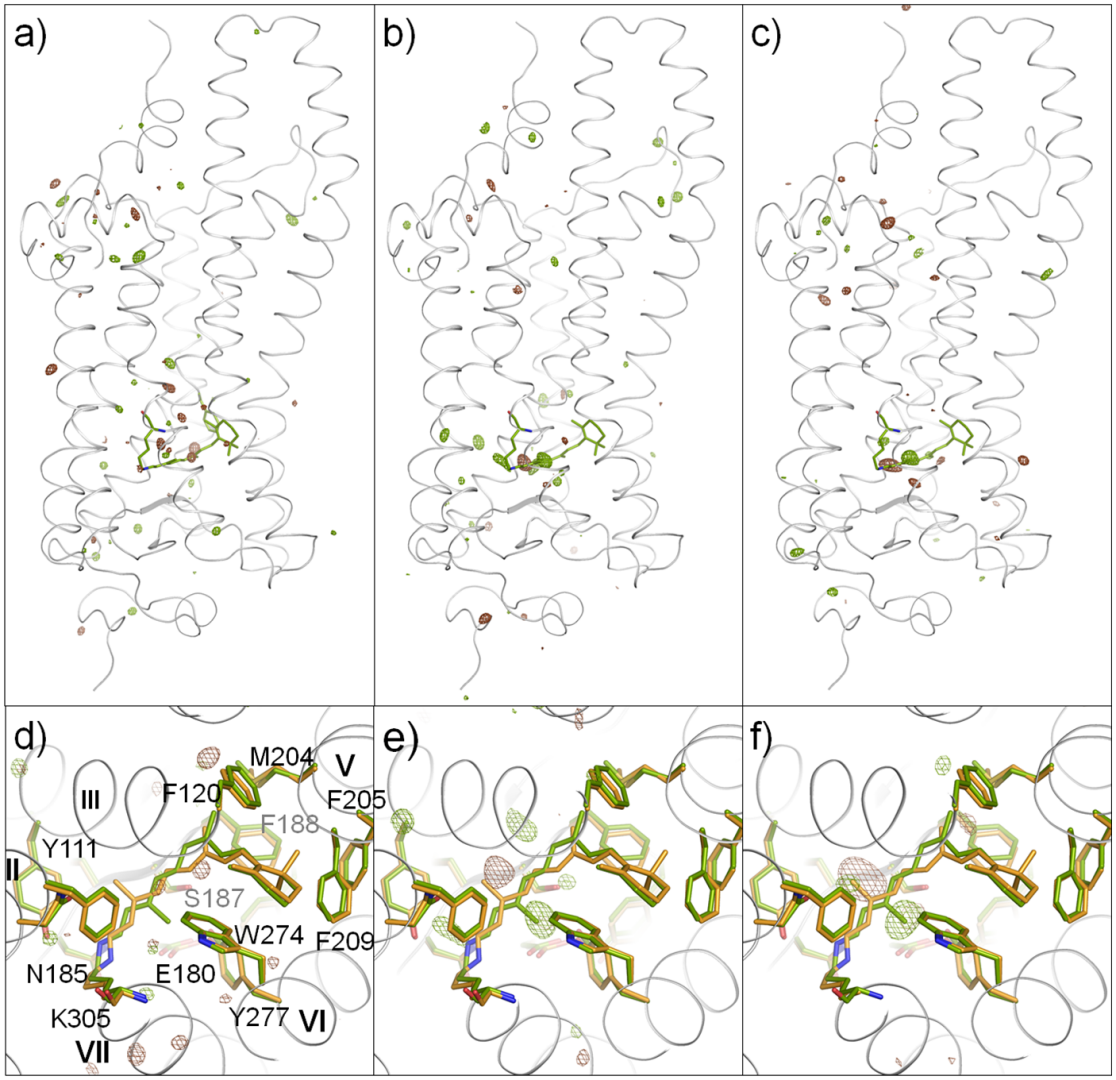
Structural difference between LUMI and ISO *versus* X-ray-induced structural change. (**a**) X-ray-induced structural change in the frozen crystals consisting of 50% LUMI, 45% ISO and 5% RHOD. This panel shows the *F*_1st_—*F*_2nd_ difference electron-density map, where *F*_1St_ is the merged diffraction data collected in an initial phase of X-ray exposure (i.e. the first 30 frames), while *F*_2nd_ is the merged diffraction data collected in a late phase of X-ray exposure (i.e. the 31st to the 60th frames). (**b**, **c**)The difference maps, Δρ_1st_(Blue/170K—Yellow) and Δρ_2nd_(Blue/170K—Yellow), derived by comparing *F*_1st_ (b) or *F*_2nd_ (c) with the diffraction amplitude from a frozen crystal consisting of 98% ISO and 2% RHOD. The maps are contoured at 3.6 σ (brown, positive density; green, negative density), respectively. White ribbon and green stick models show the structural models of ISO. (**d**—**f**) Enlarged views of the difference maps (a-c). The carbon atoms in LUMI and ISO are drawn in orange and green, respectively.

Fig 3a and 3d show the difference electron-density maps derived from *F*_1st_ and *F*_2nd_. Although the *F*_1st_—*F*_2nd_ difference map exhibited weak peaks in the vicinity of retinal, the signal-to-noise ratio was not high enough to obtain definitive information as to the structural changes induced by an X-ray flux of ~6 × 10^14^ photons/mm^2^. Nonetheless, it was suggested that the postulated structural change induced by this level of X-ray flux is restricted to the vicinity of retinal. Our previous crystallographic study of RHOD showed that X-ray-induced cleavage of the disulfide bond between Cys108 and Cys186 and decarboxylation of Asp80 become noticeable after exposure to an X-ray flux of ~ 10^16^ photons/mm^2^. In this study, diffraction data collected using a much lower flux of X-rays (6 × 10^14^ photons/mm^2^) were merged and the merged data were used for the structural analysis of LUMI.

### Structural difference between LUMI and ISO

The structural model of ISO was previously constructed using diffraction data from a frozen crystal containing ISO as the major state (98%), i.e., a crystal that was illuminated at 100 K with yellow light at 560 nm. This model was used as an initial model for the structural analysis of LUMI. In the case that there was no X-ray damage, the difference electron density map between LUMI and ISO, Δρ(lumi—iso), would be evaluated by comparing the diffraction data from a crystal containing ISO with those from crystals that had been warmed to 170 K after illumination at 100 K with blue light (Fig 3b). Strictly speaking, the difference density map derived from these diffraction data is given by: Δρ(Blue/170 K—Yellow) ≡ ρ(Blue/170 K)- ρ(Yellow) = 0.50 × ρ_lumi_—0.53 × ρ_iso_ + 0.03 × ρ_rhod_, where ρ(Blue/170K) is the electron density in the crystal that was illuminated at 100 K with blue light and transiently warmed to 170 K, and ρ(Yellow) is the electron density in the crystal that was illuminated at 100 K with yellow light; ρ_lumi_, ρ_iso_ and ρ_rhod_ are the electron densities of LUMI, ISO and RHOD, respectively. But, as the difference in the content of RHOD for the two investigated crystals was small, Δρ(Blue/170 K—Yellow) can be regarded to be nearly identical to 0.50 × Δρ_lumi-iso_.

Our major concern was the possible influence of X-ray-induced structural changes on the difference map Δρ(Blue/170K —Yellow). To investigate this influence, two difference maps, Δρ_1st_(Blue/170 K—Yellow) and Δρ_2nd_(Blue/170 K—Yellow), were evaluated using the differently merged datasets *F*_1st_ and *F*_2nd_, which were defined in the previous section. In Fig 3, panels e and f show enlarged views of the difference maps calculated using *F*_1st_ and *F*_2nd_, respectively. Although these maps are not completely identical to each other, a pair of positive and negative densities around of the C13 methyl of retinal is commonly seen in these maps. A possible interpretation of this feature is that the C13 methyl of 9-cis retinal in ISO is directed toward helix 6, whereas the C13 methyl of all-trans retinal in LUMI is directed to helix III. A peculiar feature of the map calculated using F_1st_ (Fig 3b and 3e) is the negative density near the Schiff base, though this density was less significant in the map calculated using F_2nd_ (Fig 3c and 3f). Although the latter map has a positive density around the side chain of Ser187, this peak is not clear in Fig 3a and 3d. It may be argued that this side chain moved slightly during exposure to a very low X-ray flux. In other regions of the protein, no significant changes were detected.

### Structure of LUMI

**Fig 4:**
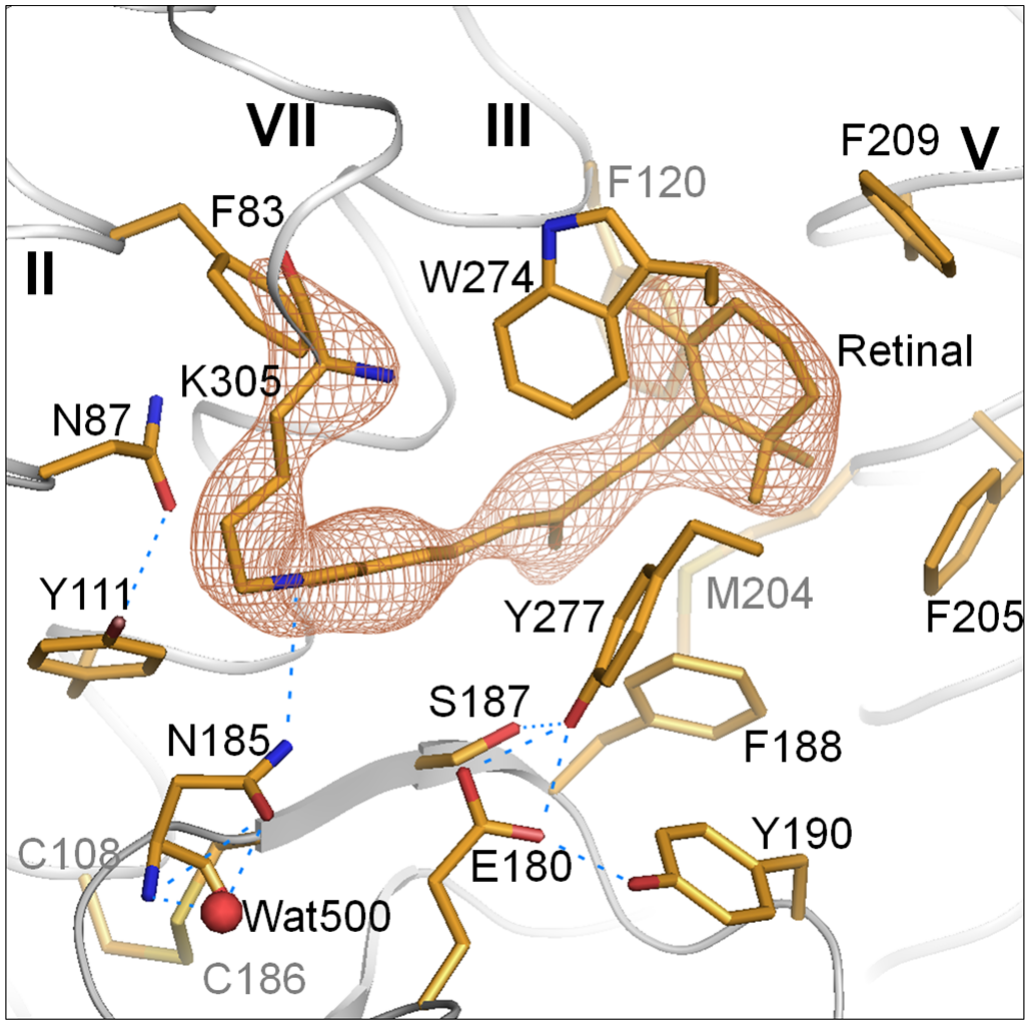
Omit map of the retinal-Lys305 chain in LUMI, contoured at 4.0 σ and overlaid on the structural model of LUMI. Oxygen, nitrogen and sulfur atoms are shown in red, blue and green, respectively.

The structural model of LUMI was first constructed to account for the difference map Δρ(Blue/170 K-Yellow/100 K) as shown in Fig 3b and 3e, and its structure was refined using the structural factor, *F*_lumi_, that was given by the following approximation: *F*_lumi_ ~ 2 × *F*(Blue/170K)—*F*(Yellow), where *F*(Blue/170 K) is the structure factor derived using the merged diffraction dataset from crystals consisting of 50% LUMI, 45% ISO and 5% RHOD, and F(Yellow) is the diffraction amplitude from a crystal consisting of 97% ISO and 3% RHOD. Although ISO was liable to X-ray radiation, the influence of its X-ray damage was removed in the derivation of *F*_lumi_ (Fig 4). It should be mentioned, however, that what we can determine based on the above approximation is the structure of a dynamic equilibrium between the undamaged LUMI and its first X-ray product(s), which is established at an X-ray flux as low as 1.6 × 10^14^ photons/mm^2^. In the following discussion, we tentatively assume that the structural difference between the undamaged LUMI and its first X-ray product(s) is very small.

**Fig 5:**
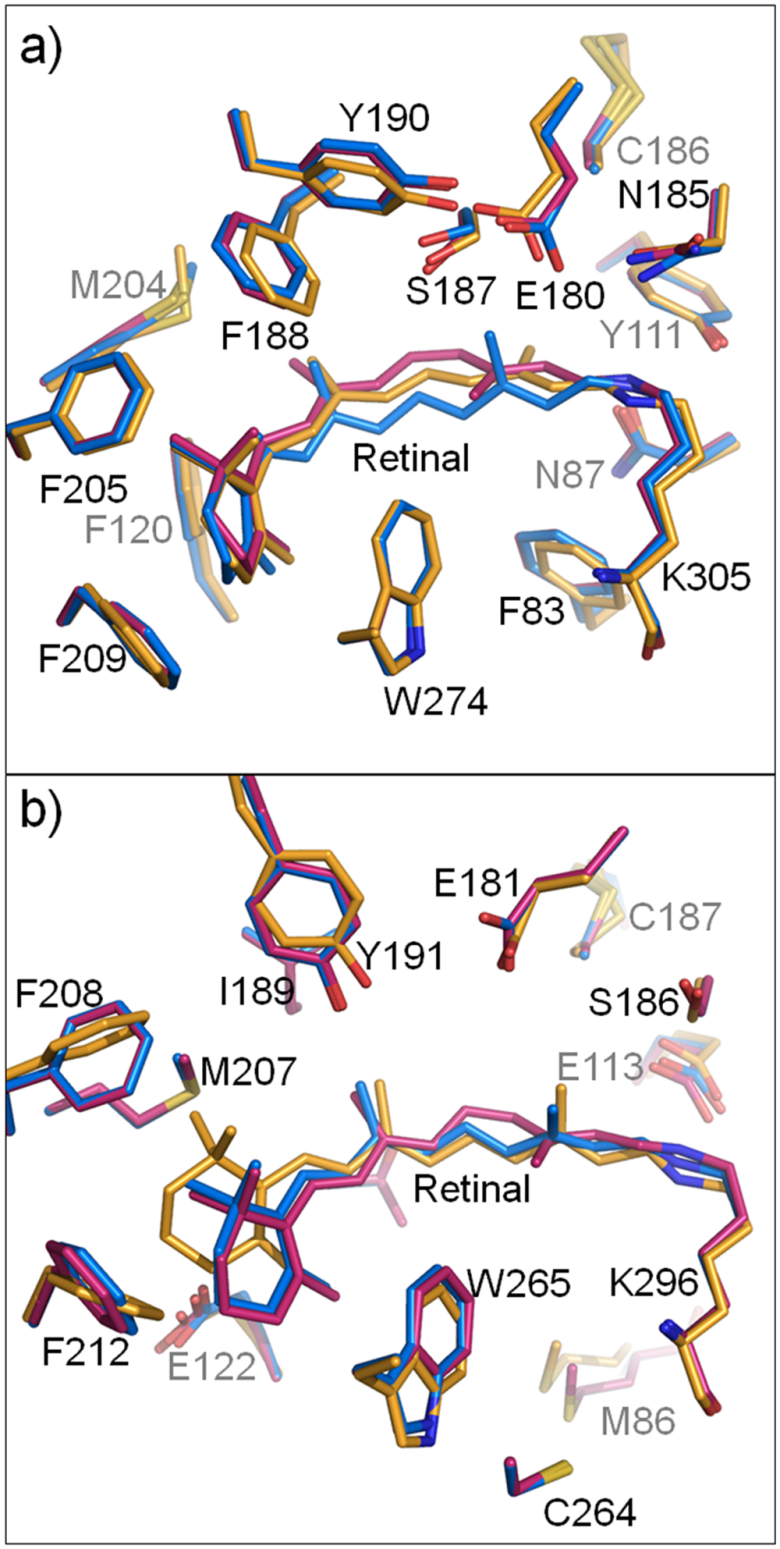
Structural changes in the early stage of the photoreaction of (a) squid rhodopsin and (b) bovine rhodopsin. Carbon atoms are shown in orange (LUMI), magenta (RHOD) and pale (BATHO). Oxygen, nitrogen and sulfur atoms are shown in red, blue and green, respectively.

In Fig 5, the structural model of LUMI was compared with those of RHOD and BATHO. It has been suggested that, due to tight contact with the surrounding phenylalanine residues, the β-ionone ring scarcely moves in the transition from RHOD through BATHO to LUMI. Although the C7 = C8 double bond and the adjacent bonds are largely twisted in BATHO, these bonds take on more relaxed configurations in LUMI. This relaxation is accompanied by a large movement of the central moiety of retinal towards the extracellular side. As a consequence, the retinal polyene chain in LUMI is sandwiched by the main chain of helix III and a β-strand in the E2 loop, as observed in RHOD (and ISO). It is noteworthy that the longitudinal length of the retinal from the β-ionone ring to the Schiff base remains unchanged throughout RHOD, BATHO and LUMI. Indeed, the all-trans retinal in LUMI is gently curved so as to embrace the indole ring of Trp274.

The most significant difference between LUMI and RHOD is seen in the orientation of the C13 methyl group; i.e., it points toward helix VI in RHOD, whereas it points towards helix III in LUMI. This alteration is accompanied by reorientation of the Schiff base NH bond; i.e., it is directed towards Asn87 of helix II in RHOD, whereas it is re-orientated towards Asn185 in LUMI (Fig 4).

## Discussion

A previous crystallographic study of squid rhodopsin showed that the all-trans retinal in BATHO is largely twisted in such a manner that the C13 methyl is directed to Ser187 and pushes its side chain [7]. It is shown here that the BATHO-to-LUMI transition is accompanied by re-orientation of the C13 methyl toward helix III, which can be accomplished by overcoming the steric conflict with the side chain of Ser187. After this transition, the polyene chain assumes a less distorted planar configuration and the center of the gravity of the retinal polyene chain returns to the same position as seen in RHOD and (Fig 4). It can be argued that the distortion energy stored around the central part of retinal in BATHO is transferred to the distortion energy around the retinal Schiff base, the hydrogen-bonding partner of which is suggested to be switched from Asn87 to Asn185 upon the formation of LUMI. This argument is in line with the results of a previous FTIR study of octopus rhodopsin, which showed that the structure around the Schiff base is distorted in LUMI [19]. Needless to say, elucidation of the detail of this distortion is crucial for better understanding of the activation mechanism of squid rhodopsin. However, as the structure around the Schiff base seemed to be modified by a very low X-ray flux, it is currently difficult to obtain definite information about the interaction mode between the Schiff base and the nearby residue Asn185 or the counter-ion Asp180. We need to overcome this difficulty for a quantitative discussion about the accuracy of the retinal bond twists, which has been a matter of debate.

In Fig 5, the structural changes occurring in the early stage of the photoreaction of squid rhodopsin are compared with those reported for bovine rhodopsin. A peculiar feature of the photoreaction of squid rhodopsin is seen in that the position/orientation of the β-ionone ring of retinal, which is tightly fixed by the surrounding aromatic residues, remains unaltered upon the formation of LUMI. This observation agrees with the results of a previous mutational study of amphioxus rhodopsin, which showed that the β-ionone ring of retinal keeps its position until the active state is generated [20]. In bovine rhodopsin, on the other hand, the β-ionone ring moves largely towards helix IV upon the formation of LUMI [11]. In the active state of bovine rhodopsin, the β-ionone ring is further far away from the original position [12]. Such a movement of the β-ionone ring may be allowed in bovine rhodopsin, which possesses a hydrophilic residue (Glu122) near the β-ionone ring.

When the structural differences between squid rhodopsin and bovine rhodopsin are taken into account, it may be more accurate to postulate that the details of their activation mechanisms are different from each other. Indeed, bovine rhodopsin is hydrolyzed to free retinal and apoprotein after photo-activation, whereas the Meta state of squid rhodopsin is thermally stable [1]. It is noteworthy that whereas signal transduction in vertebrate vision is mediated by the second messenger cyclic GMP, signal transduction in squid photoreceptor cells uses an inositol-1,4,5-trisphosphate signaling cascade in which photoactivated rhodopsin stimulates a G_q_-type G protein. As the latter is used by numerous GPCRs, the structural data of the photoreaction states of squid rhodopsin would provide insights into the activation mechanism of other G_q_-type GPCRs.

## Materials and Methods

### Protein purification and crystallization

All manipulations were performed in dim red light (>640 nm). Squid (*Todarodes pacificus*) rhodopsin contains 448 amino acids with a molecular mass of 50 kDa. C-terminally truncated squid rhodopsin was crystallized as described previously [20]. Briefly, the extension in the C-terminal region was removed by cleaving a peptide bond at Glu373 (or Glu 358) with V8 protease. The truncated rhodopsin was selectively extracted from microvillar membranes with octylglucoside in the presence of zinc acetate and crystallized into the hexagonal *P*6_2_ crystal. The present work does not involve experimentation on living vertebrates, therefore, no permission was required from the animal ethics committee of Nagoya university.

### Measurements of absorption spectra

Absorption spectra of a frozen crystal of squid rhodopsin were measured using a microspectrophotometer in which monochromatic light from a double monochromator (Shimadzu UV350A) was focused on a small area of the crystal, and the intensity of transmitted light was measured using a photomultiplier tube, as described previously [9,21]. A frozen crystal was mounted onto a goniometer head attached to the microspectrophotometer and the temperature was controlled by a flow of cold nitrogen gas from a cryostreamer (Oxford Cryosystems, CC-12). For absorption measurements of the unphotolyzed state of squid rhodopsin, crystal mounting was performed in dim red light. For absorption measurements of the BATHO and ISO states of squid rhodopsin, the frozen crystal was irradiated at 100 K for 3 minutes with monochromatic light from a blue laser (λ = 473 nm, 2 mW/mm^2^) or monochromatic light that was obtained by passing white light from a tungsten lamp (150W) through an interference filter of 560 nm (Toshiba). For investigation of the BATHO-to-LUMI transition in a crystal at various temperatures, the frozen crystal that was irradiated at 100 K with the blue laser was warmed in the dark by controlling the temperature of the cold nitrogen gas.

### Data collection

A single crystal was transferred into a solution containing 30 m*M* octylglucoside, 3.2 *M* ammonium sulfate, 40 m*M* MES pH 6.4 and 20% sucrose. After soaking for 10 min, the crystal was flash-frozen in liquid propane held at its melting temperature and stored in liquid nitrogen. For preparation of a mixture state of LUMI, RHOD and ISO, a frozen crystal at 100 K was illuminated for 1 minute with blue light to accumulate BATHO. Then, the crystal temperature was increased to 170 K in the dark to facilitate the BATHO-to-LUMI transition, and re-cooled to 100 K. X-ray diffraction measurements were performed on SPring-8 BL38B1, where a frozen crystal kept at 100 K was exposed to a monochromatic X-ray beam at a wavelength of 1.0 Å. Indexing and integration of diffraction spots were carried out with *Mosflm* 7.04 [22]. Diffraction data from the hexagonal crystal were fitted well by the unit-cell parameters described in Table 1. The scaling of data was carried out using *SCALA* [23] in the *CCP4* program suite [24]. Diffraction data from partially twinned crystals were de-twinned with *CCP4*.

**Table 1:** Data collection and final refinement statistics.

Data collection	LUMI
Space group	*P6*_*2*_
Cell dimensions	
*a*, *b*, *c* (Å) α, β, γ (°)	122.39,122.39,158.77 90, 90, 120
Twinning fraction (%)	13
Resolution (Å)	44.07–2.80 (2.95–2.80)
*R*_sym_ or *R*_merge_	0.133 (0.494)
*I* / σ*I*	7.1 (2.7)
Completeness (%)	85.9 (91.4)
Redundancy	4.2 (4.0)
**Refinement**	
Resolution (Å)	44.0–2.8
No. reflections	27873
*R*_work_ / *R*_free_	0.289 / 0.325
Protein atoms	5839
No. lipid molecules	7
No. Water	59
R.m.s. deviations	
Bond lengths (Å)	0.09
Bond angles (°)	1.4

### Structure refinement

Structure refinement was done with *CNS* 1.3 [25, 26].and *XtalView* 4.1 [27]. Model building was performed by the molecular-replacement method with the structure of squid isorhodopsin (Protein Data Bank accession 3AYN) as an initial search model. All figures were generated using *PyMOL* [28].
